# Drugs in dental biofilm and enamel – A pilot study

**DOI:** 10.1016/j.heliyon.2023.e23177

**Published:** 2023-12-02

**Authors:** Alexander Riedel, Merja A. Neukamm, Miriam Klima, Kerstin Henkel, Volker Auwärter, Markus J. Altenburger

**Affiliations:** a Department of Operative Dentistry and Periodontology, Center for Dental Medicine, Medical Center – University of Freiburg, Hugstetter Str. 55, Freiburg im Breisgau, DE, 79106, Germany; b Faculty of Medicine, University of Freiburg, Breisacher Str. 153, Freiburg im Breisgau, DE, 79110, Germany; c Institute of Forensic Medicine, Forensic Toxicology, Medical Center – University of Freiburg, Albertstr. 9, Freiburg im Breisgau, DE, 79104, Germany; d Labor Berlin – Charité Vivantes GmbH, Department of Laboratory Medicine and Toxicology, Berlin, Sylter Str. 2, Berlin, DE, 13353, Germany

**Keywords:** Teeth, Oral cavity, Plaque, LC-MS/MS, Forensic toxicology

## Abstract

**Objective:**

Enamel and dental biofilm might serve as alternative matrices for determination of illicit and medical drugs. Thus, this study aims at evaluating possible correlations between detected drug concentrations in the matrices and simulated drug use in situ.

**Design:**

Eleven subjects wore intraoral splints with embedded demineralized bovine enamel samples. Drug use was simulated by mouth rinsing with a 1.0 μg/ml drug solution three times daily for 1 min (study A) or by incubation of the splints in a 10 μg/ml drug solution once a day for 30 min (study B). Amphetamines, opiates, cocaine and benzoylecgonine were used as drugs. After 11 days, biofilm and enamel samples of the intraoral splints were analyzed by liquid chromatography mass spectrometry after drying and extraction via ultrasonication with acetonitrile (biofilm) or methanol (enamel).

**Results:**

In study A, median and mean drug concentration ± standard deviation were 1.3 pg/mg and 6.4 ± 11 pg/mg in biofilm and 0.2 pg/mg and 0.5 ± 0.9 pg/mg in enamel. In study B, median and mean drug concentration ± standard deviation were 350 pg/mg and 1100 ± 1600 pg/mg in biofilm and 5.8 pg/mg and 9.9 ± 10 pg/mg in enamel.

**Conclusions:**

Overall, there were considerable interindividual concentration differences. Correlations between concentrations in the two sample materials were shown. The results of this pilot study revealed a dependence of concentrations on intensity and duration of drug contact. Thus, important information on past drug use might be provided in forensic cases by analysis of dental biofilm and enamel.

## Highlights

• Determination of amphetamines, opiates and cocaine in enamel and dental biofilm.
• Detection of drugs by analysis with liquid chromatography mass spectrometry.
• Drug concentrations depend on intensity and duration of drug contact.
• Deposited biofilm has a major impact on drug incorporation into enamel.
• Teeth and plaque serve as sensitive alternative matrices in forensic toxicology.

## 1 Introduction

Today, opioids are an indispensable part of pain treatment, but their medical use can lead to abuse. In addition to opioids, illicit drugs such as cocaine, amphetamine and ecstasy (MDMA) are part of the current drug market [1]. Research on alternative matrices is important to improve the quality of drug detection, especially if other matrices are not available. For example, dental hard tissues can be used for analysis in cases of burnt, putrefied or skeletonized remains [2]. After consumption, drugs pass into oral fluid [[3], [4], [5]] and get incorporated in enamel during remineralization. Klima et al. [6,7] investigated the incorporation of illicit and medical drugs in different dental hard tissues in vitro and postmortem. Dental hard tissues were used to determine metal exposition [8], but also alcohol and nicotine consumption [9,10]. Furthermore, oral fluid is in contact with dental biofilm. Therefore, drugs of abuse can be detected by analyzing dental biofilm samples [11]. Since positive findings in tooth hard substances might indicate drug use dating back longer and dental biofilm can be used to determine recent drug use, the simultaneous analysis of both matrices provides a broad window of information [7,11,12]. It has been shown that the pellicle has a protective effect against wear and erosive changes on the enamel surface [[13], [14], [15]]. Thus, the organic deposition on the dental surface could build a barrier for drugs and reduce incorporation in enamel. As an opposing point of view, dental biofilm could act as a kind of reservoir, enabling the transfer of drugs to enamel. Such depot effects for drugs were observed in the oral mucosa [16], but also for chlorhexidine in dental pellicle [17] and for fluoride in the tongue coating [18]. However, presently, the authors are not aware of studies that show a correlation between drug incorporation into dental biofilm and enamel. The present in situ study deals with the detection of amphetamine, methamphetamine, 3,4-methylenedioxy-N-methylamphetamine (MDMA), 3,4-methylenedioxy-N-ethylamphetamine (MDEA), 3,4-methylenedioxyamphetamine (MDA), morphine, codeine, 6-monoacetylmorphine (6-MAM), cocaine and benzoylecgonine (BE) in dental biofilm and bovine enamel after treatment with a respective drug solution. Based on detected concentrations, the suitability of biofilm and enamel for determination of drugs of abuse as well as interindividual differences and differences in the incorporation of the drugs were to be examined. In addition, correlations between drug concentrations in biofilm and enamel should be evaluated and other influencing factors for drug incorporation like intensity and duration of drug contact should be identified.

## 2 Materials and methods

### 2.1 Subjects

The study was performed with eleven healthy subjects of which nine were female and two male, aged between 21 and 48 years. Any contact with drugs of abuse in the past 6 months and a current pregnancy disqualified subjects for participation. The study was approved by the BfArM (Federal Institute for Medical Drugs and Medical Devices) and the ethics committee of the University of Freiburg (Reference Number: EK 334/14). All subjects gave their written consent and signed a privacy statement.

### 2.2 Intraoral splints

**Fig. 1:**
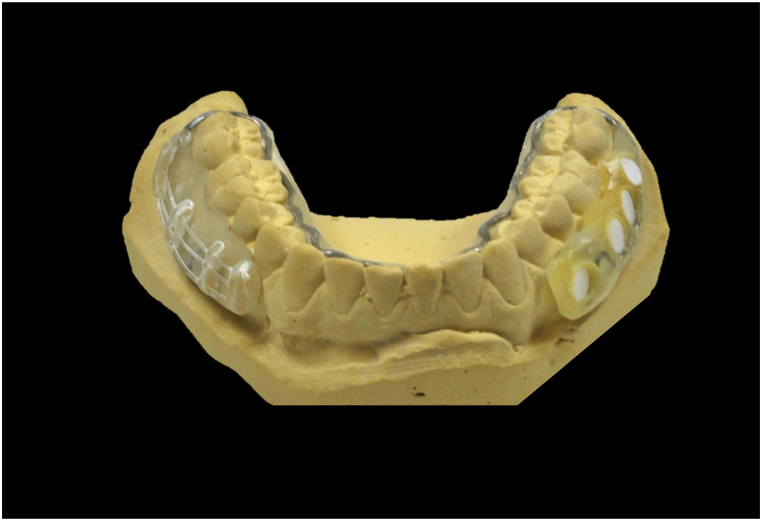
Splint design and its position on a model of the lower jaw.

Individual intraoral splints in the lower jaw, made of acrylic resin (Orthocryl, Dentaurum, Ispringen, Germany) and stabilized with wire (Wipla, Dentsply Sirona, Konstanz, Germany), were used as sample holders. A total of four demineralized bovine enamel samples were embedded in the vestibular sides of the buccal aspects of the splints. Enamel samples were demineralized according to Buskes et al. [19] and had a mineral loss of at least 1500 vol.-% x μm. Additional to biofilm formation on the enamel surfaces, nylon threads (Hobbygross Erler, Rohrbach, Germany) were applied on the vestibular sides of the buccal aspects of the splints to improve biofilm formation by increasing biofilm adherence and reducing its removal. Fig. 1 shows the design of the splints and their position on a model of the lower jaw.

### 2.3 Mouth rinse and incubation drug solutions

Amphetamine, methamphetamine, MDMA, MDEA, MDA, morphine, codeine, 6-MAM, cocaine and BE (Lipomed, Weil am Rhein, Germany) were dissolved in a solution of 0.9 % NaCl (B. Braun, Melsungen, Germany) and diluted. The mouth rinse solution in study A had a concentration of 1.0 μg/ml. The incubation drug solution in study B had a concentration of 10 μg/ml.

### 2.4 Calibration solutions and internal standard solution

Methanolic reference solutions (1 μg/ml) containing amphetamine, methamphetamine, MDMA, MDEA, MDA, morphine, codeine, 6-MAM, cocaine and BE (LGC Standards, Wesel, Germany) were diluted with methanol (Sigma-Aldrich, Steinheim, Germany) and used for calibration. Deuterated derivatives of all drugs (LGC Standards, Wesel, Germany) were used as internal standards. The concentration of the internal standard solution was 50 ng/ml in methanol.

### 2.5 Solvent A/B for analysis with LC-MS/MS

1 l of solvent A contained 1 ml formic acid (AppliChem, Darmstadt, Germany) and 100 μl solution of ammonium formate (Sigma-Aldrich, Steinheim, Germany) in deionized water. 1 l of solvent B contained 1 ml formic acid in methanol.

### 2.6 Course of the study

The present in situ study included two parts, each conducted in 11 days. Drug contact of biofilm and enamel in study A was performed by using the mouth rinse drug solution (1.0 μg/ml) three times daily for 1 min. In study B, an increased intensity of drug contact was simulated by incubation of the splints in the higher concentrated drug solution (10 μg/ml) once a day for 30 min. Prior to drug contact, all subjects wore the intraoral splints for 7 days in both parts of the study to obtain adequate amounts of biofilm for drug incorporation at the beginning of the respective study. Furthermore, biofilm on the teeth of the subjects was collected prior to drug contact. These samples served as reference for analysis. Cleaning of the vestibular sides of the splints was not allowed to protect the samples. General oral hygiene could be continued as usual. However, antimicrobial agents were not allowed. Sample collection in study A and B took place 4 h after the last drug contact. The study was conducted on premises of the University of Freiburg.

### 2.7 Sample preparation and analysis

Preparation of the samples and following analysis with LC-MS/MS was based on validated methods of Henkel et al. [11] and Spinner et al. [20]. For analysis, biofilm on the enamel surfaces was collected together with biofilm on the splints and dried for 4 h at room temperature. Enamel samples were pulverized in a ball mill (MM 400, Retsch, Haan, Germany) after drying in the oven (B5050E, Heraeus, Hanau, Germany) for 24 h at 37 °C. Due to low expected concentrations in study A, all four enamel samples of one subject were pooled prior to sample preparation. In study B, enamel samples were analyzed separately. All samples were weighted after drying. The whole sample material was used for analysis. 10 μl of the internal standard solution were added to each biofilm and enamel sample. Drugs were extracted from biofilm with 500 μl acetonitrile (VWR International, Fontenay-sous-Bois, France) in an ultrasonic bath (Emmi-H22, EMAG, Mörfelden-Walldorf, Germany) for 10 min. Extraction from enamel was performed three times with 500 μl methanol in an ultrasonic bath for 60 min. After centrifugation (Heraeus Megafuge 1.0, Thermo Fisher Scientific, Schwerte, Germany) for 10 min at 16100 x *g*, the supernatants were transferred to glass vials (WICOM, Heppenheim, Germany) and reduced under a gentle stream of nitrogen at 40 °C to a small residual volume. After addition of 100 μl 2-propanol (Carl Roth, Karlsruhe, Germany) / hydrochloride (VWR International, Fontenay-sous-Bois, France) (3/1, v/v) to prevent evaporation of amphetamines, the extract was evaporated to dryness. The dry residue was reconstituted in 100 μl solvent A/B (95/5, v/v). All samples were analyzed with LC-MS/MS in scheduled multiple reaction monitoring (sMRM) mode, recording two characteristic mass transitions per analyte. A Shimadzu UHPLC (Shimadzu, Duisburg, Germany) and a Qtrap 5500 triple-quadrupol-MS/MS (AB Sciex, Darmstadt, Germany) were used to analyze biofilm. For enamel analysis, a Dionex UltiMate 3000 HPLC (Thermo Fisher Scientific, Dreieich, Germany) and an API 5000 triple-quadrupol (AB Sciex, Darmstadt, Germany) was used. The software Analyst 1.6 (AB Sciex, Darmstadt, Germany) was used to operate the LC-MS/MS and for data analysis. A calibration range from 5 to 1500 pg/mg was applied for biofilm and from 0.1 to 20 pg/mg for enamel. Lower limits of quantification were at the respective lower limit of the calibration range. Limits of detection were estimated at a signal noise ratio of 3:1. Statistical analysis was performed with Stata 16.1 (StataCorp LLC, College Station, USA) using a significance level of < 0.05. Equipment for sample collection included widely available basic dentistry tools. Analyses can be performed in the toxicological laboratory of medicolegal institutes or samples can be sent to other respective laboratories, depending on individual conditions also adapted to the individual situation.

## 3 Results

Twelve volunteers were screened out of which eleven were included into the study. All participants completed the studies A and B according to the study protocol. No adverse events or adverse drug reactions occurred. Limits of detection for biofilm were between 1.0 and 5.0 pg/mg for all drugs except MDA with 20 pg/mg. Limits of detection for enamel were between 0.04 and 0.1 pg/mg for all drugs except amphetamine with 0.4 pg/mg and MDA with 0.2 pg/mg.

### 3.1 Study A: Mouth rinse with drug solution (1.0 μg/ml) three times daily for 1 min

**Fig. 2:**
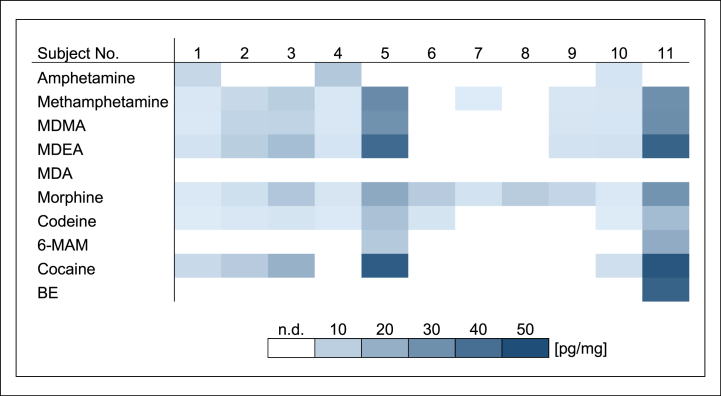
Drug concentrations in dental biofilm in study A [pg/mg] (mouth rinse (1.0 μg/ml) three times daily for 1 min).

**Fig. 3:**
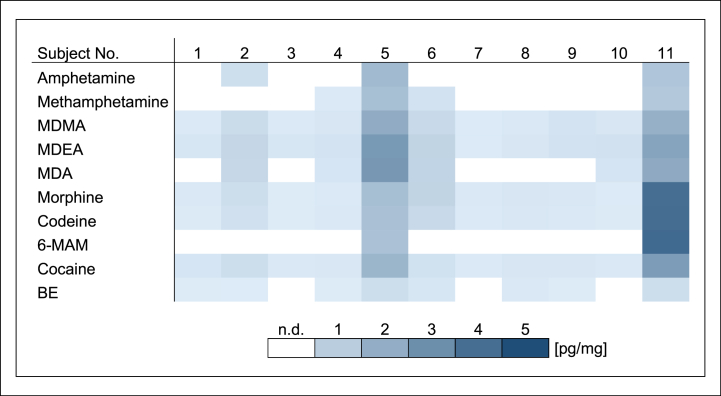
Drug concentrations in demineralized enamel samples in study A [pg/mg] (mouth rinse (1.0 μg/ml) three times daily for 1 min).

The biofilm samples taken prior to drug contact were negative for all substances. In biofilm collected after the last drug contact, all drugs except MDA could be detected, but not in every sample. Drug concentrations in biofilm ranged from 1.3 to 48 pg/mg (Fig. 2). Considerable concentration differences were observed between both, subjects and drugs. Subject 5 and 11 stood out clearly due to high concentrations and increased numbers of positive drug findings. In enamel, all drugs including MDA could be determined, but as in biofilm not in every sample. Drug concentrations in enamel ranged from 0.04 to 4.1 pg/mg (Fig. 3). Detected drug concentrations and interindividual concentration ranges in enamel were substantially lower than those in biofilm. There were high drug concentrations in samples of subject 5 and 11 as well and all drugs could be detected in these enamel samples.

### 3.2 Study B: Incubation in drug solution (10 μg/ml) once a day for 30 min

**Fig. 4:**
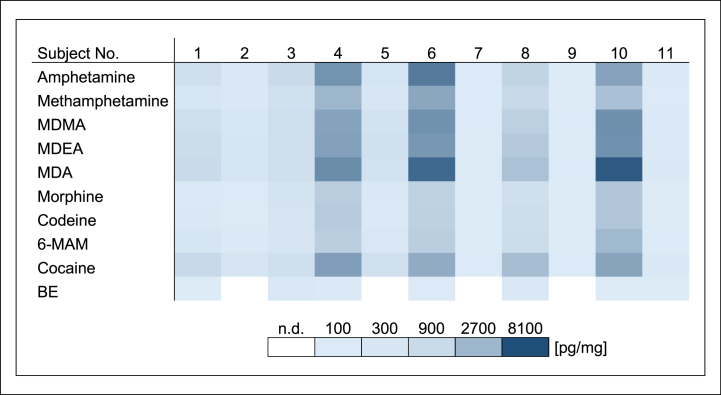
Drug concentrations in dental biofilm in study B [pg/mg] (incubation solution (10 μg/ml) once a day for 30 min).

**Fig. 5:**
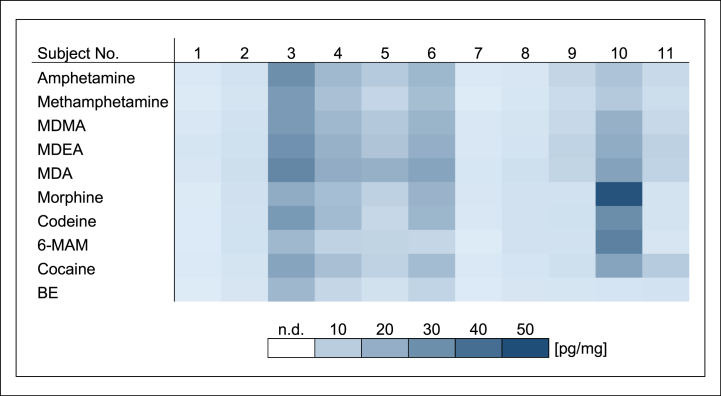
Drug concentrations in demineralized enamel samples (mean of all four samples) in study B [pg/mg] (incubation solution (10 μg/ml) once a day for 30 min).

The biofilm samples taken prior to drug contact were negative for all substances. In biofilm collected after the last drug contact, all drugs except BE could be detected in every sample. Detected drug concentrations in biofilm ranged from 6.4 to 7500 pg/mg (Fig. 4). Considerable concentration differences were also observed between both, subjects and drugs. Subject 4, 6 and 10 had substantially higher concentrations. Comparison of the single samples showed increased concentrations of amphetamines and cocaine, whereas concentrations of opiates and BE were lower in general. In enamel, all drugs could be detected in most of the samples. Detected drug concentrations in enamel ranged from 0.2 to 65 pg/mg. Mean concentrations of all four samples of one subject are shown in Fig. 5. Detected concentrations and interindividual concentration ranges were considerably lower than those in biofilm. There were high drug concentrations in samples of subject 4, 6 and 10 just like in the respective biofilm samples. Especially, the morphine findings in enamel samples of subject 10 were conspicuously high. Unexpectedly, subject 3 had high concentrations of the drugs in enamel, but only average concentrations in biofilm. Overall, concentration differences between the drugs were lower in enamel than in biofilm. The highest concentrations were observed for MDA.

### 3.3 Correlation between concentrations in biofilm and enamel

Subjects with high drug concentrations in biofilm had also increased concentrations in enamel samples in both, study A and B. Because of the high number of negative findings in study A, linear regression analyses for statistical assessment of this correlation were only performed on the results of study B. There was a significant linear relation (p < 0.05) between biofilm and enamel concentrations of morphine, codeine, 6-MAM and BE. Further linear regression analyses were performed without the outlying results of subject 3, having very high concentrations in enamel and low concentrations in biofilm. The results showed a significant linear relation (p < 0.05) for all drugs.

### 3.4 Comparison of study A and B

Overall, considerable differences were observed between both, the sample materials and the two study parts. Median and mean drug concentrations as well as standard deviations of all biofilm samples and all enamel samples in study A and B are shown in Table 1. After incubation in a 10 μg/ml drug solution for 30 min once a day in study B, detected drug concentrations and number of positive findings in biofilm and enamel were clearly increased in comparison to mouth rinse with a 1.0 μg/ml drug solution for 1 min three times daily in study A (see Fig. 2, Fig. 3, Fig. 4, Fig. 5). Biofilm had remarkably higher drug concentrations than the respective enamel samples in both studies. In addition, high interindividual differences in biofilm formation were observed in both, study A and study B. Biofilm sample weights ranged between 0.28 and 4.12 mg.

**Table 1:** Median concentrations, mean concentrations and standard deviations of all biofilm samples and all enamel samples in study A and B [pg/mg].

	Study A		Study B	
	Biofilm	Enamel	Biofilm	Enamel
Median concentration [pg/mg]	1.3	0.2	350	5.8
Mean concentration [pg/mg]	6.4	0.5	1100	9.9
Standard deviation [pg/mg]	11	0.9	1600	10

## 4 Discussion

### 4.1 Determination of drugs in dental biofilm

The results showed that biofilm can be used for determination of drugs of abuse. However, considerable concentration differences were observed between the single subjects, although both studies were performed under standardized conditions with regard to defined contact times and uniform concentrations of the drug solution. This observation could be explained by an individual formation of voids and channels in biofilms leading to a complex and heterogeneous individual biofilm structure [[21], [22], [23]]. In addition, the diversity of biofilm composition could play a role. For example, caries and its acid environment change the microbial community. Fatty acid methyl ester profiles can be used to characterize microbial communities [[24], [25], [26]]. At the time of writing, the authors were not aware of any studies that show a relation between the individual microbial composition of biofilms and biofilm drug concentrations. An influence on retention of drugs in dental biofilms should be considered. Furthermore, irregular distribution of the drug solution in the oral cavity during mouth rinsing has to be taken into account in study A.

### 4.2 Determination of drugs in enamel

The results showed that enamel can be used for determination of drugs of abuse. Even at lower intensity of drug contact (study A), drugs were still detectable in many cases. Detected enamel drug concentrations were low compared to biofilm concentrations and interindividual concentration ranges were lower than those in biofilm as well. This observation can be explained by differences in structure and drug incorporation. It can be assumed that oral biofilm incorporates drugs well due to its complex structure which contains numerous voids and channels [21,22,27]. However, these characteristics possibly lead to a wash out by natural salivation. In contrast, drugs get incorporated in enamel during remineralization [6].

### 4.3 Incorporation of different drugs

Concentration differences between various drugs were observed in the samples despite uniform drug concentrations in the solution. However, these differences were lower than those between single subjects (see Fig. 4, Fig. 5). The higher limit of detection for MDA could explain why it was not detected in biofilm in study A. Increased morphine concentrations in enamel samples of subject 10 in study B may be explained by an accidental consumption of poppy, as positive findings for opiates in oral fluid were associated with intake of poppy seed [16,28]. Concentrations of amphetamines and cocaine in biofilm in study B were higher than concentrations of opiates and BE, which could be due to differing molecular sizes of the drugs. The polar surface area of amphetamines is only half that of opiates. Therefore, amphetamines are potentially better incorporated into biofilm. The polar surface areas of cocaine and opiates are comparable, but the biofilm incorporation of cocaine is higher, suggesting that molecular size may not be the only influencing factor. Specific interactions between drug molecules and biofilm components could also play a role. While considerable concentration differences between different drugs were observed in biofilm, respective differences were lower in enamel. Ottaviani et al. [29] analyzed extracted teeth from drug addicts and determined lower concentrations of BE than these of cocaine. Klima et al. [6] observed an increased incorporation of amphetamines in comparison to opiates and cocaine in vitro. Therefore, they assumed that drug incorporation in dental hard tissues depends on physiochemical properties of the substances. In enamel, interactions and bonding with hydroxyapatite could be decisive for drug specific concentrations. Thus, drugs that have strong interactions in biofilm do not necessarily have strong bondings in enamel. Even though an increased incorporation in enamel was observed for MDA, not all amphetamines were better incorporated than opiates and cocaine. Overall, lower molecular size and higher polarity of a compound seems to enhance its incorporation in both, biofilm and enamel.

### 4.4 Correlation between concentrations in biofilm and enamel

Based on detected concentrations in both studies, a correlation between drug concentrations in biofilm and enamel seems conceivable. Drugs probably are mainly incorporated in enamel via remineralization and drug reservoirs in dental biofilm, rather than through direct contact. Because of defined contact times and use of demineralized bovine enamel samples for all subjects, interindividual concentration differences in enamel could be explained by different biofilm depot effects due to its individual structure and composition [[21], [22], [23], [24]]. Biofilm is in direct contact with enamel and enables communication between oral environment and enamel surface at the same time [23]. Consequently, detected drug concentrations in enamel could depend on biofilm drug concentrations. In addition to this depot effect, individual remineralization potentially influences drug incorporation in enamel. Due to the experimental setup used in this pilot study, only drug incorporation via direct contact and remineralization was investigated. Incorporation via blood could be the subject of further research. However, in this case the investigation of dentine would be more appropriate. Linear regression analyses of the results of study B (without subject 3) revealed a significant linear relation between biofilm and enamel concentrations for all drugs. The outlying biofilm concentrations of subject 3 could be explained by a damage of biofilm at the end of the incubation period. In this study, biofilm which was formed on the enamel surfaces was pooled with biofilm on the splints, which was necessary to get enough sample material for analysis. This could be a limiting factor as the interpretation of authentic correlations requires the assumption that the pooled biofilm is representative for authentic biofilm. Another limiting factor is that authentic samples of drug addicts cannot be used as a standardization of the course of the study is not possible and it is impossible to force someone to use drugs in a controlled manner. Moreover, authentic enamel removal is invasive and irreversible. This correlation could be important to understand the process of drug incorporation into biofilm and enamel.

### 4.5 Other possible influencing factors for drug incorporation

There were high interindividual differences in biofilm sample weights. Based on individually differing amounts of formed plaque, differentiation between heavy and light plaque formers is possible [23,30]. Biofilm structure and composition could depend on the type of plaque formation, too. Therefore, an influence of the amount of formed biofilm on drug incorporation in the samples is conceivable. However, the present study did not show an appropriate relation. The size of biofilm surfaces could be important in explaining incorporation, as a large surface entails a big contact zone between drug solution and biofilm, potentially resulting in an enhanced incorporation. Influences of biofilm surface sizes could be the subject of further research.

Nutrition could influence drug concentrations in biofilm and enamel as well, because individual remineralization depends on the type and frequency of food intake [3,14,15]. For example, an industrialized western died with high processed food can be differentiated from a plant-based died [31]. However, a rough classification regarding these criteria did not reveal correlations to drug incorporation. In this context, potential lack of compliance of the participants has to be considered [32].

## 5 Conclusions

Analysis of drug concentrations in biofilm and enamel revealed considerable differences between single subjects. Comparing concentrations of the drugs, differences in incorporation were observed as well, but at a substantially lower level than the interindividual differences. Drug concentrations were significantly higher in biofilm than in enamel. Extended contact times with a higher concentrated drug solution in study B led to significant increased drug concentrations in both sample matrices. Therefore, it can be assumed that concentrations in biofilm and enamel depend on intensity and duration of drug contact. However, even short contact times with a low concentrated drug solution in study A allowed detection of most drugs, making dental biofilm and enamel suitable and sensitive matrices for drug detection. Furthermore, correlations between drug concentrations in biofilm and enamel were revealed which indicates that the deposited biofilm has a major impact on drug incorporation into enamel. More detailed information about recent drug contact can be obtained by analyzing these matrices. In addition to the postmortem application of biofilm and enamel samples, biofilm could be used in traffic controls, because of its simple and non-invasive sample collection. The hypotheses of this pilot study open up the possibility to interpret drug findings in dental biofilm and enamel.

## Data availability statement

The data are included in the article and supplementary material. They have not been deposited into a publicly available repository.

## CRediT authorship contribution statement

**Alexander Riedel** contributed to Conceptualization, Data curation, Formal analysis, Investigation, Methodology, Project administration, Software, Supervision, Validation, Visualization, Writing - original draft.

**Merja A. Neukamm** contributed to Conceptualization, Data curation, Formal analysis, Funding acquisition, Investigation, Methodology, Project administration, Resources, Software, Supervision, Validation, Visualization, Writing - review and editing.

**Miriam Klima** contributed to Data curation, Formal analysis, Investigation, Methodology, Software, Validation, Writing - review and editing.

**Kerstin Henkel** contributed to Data curation, Formal analysis, Investigation, Methodology, Software, Validation, Writing - review and editing.

**Volker Auwärter** contributed to Data curation, Formal analysis, Investigation, Methodology, Software, Writing - review and editing.

**Markus J. Altenburger** contributed to Conceptualization, Data curation, Formal analysis, Funding acquisition, Investigation, Methodology, Project administration, Resources, Software, Supervision, Validation, Visualization, Writing - review and editing.

All authors gave their final approval and agree to be accountable for all aspects of the work ensuring integrity and accuracy.

## Declaration of competing interest

The authors declare that they have no known competing financial interests or personal relationships that could have appeared to influence the work reported in this paper.
